# Synchronizing sucrose effluxers with influxers in phloem loading for yield output and adaptation to environment

**DOI:** 10.1093/nsr/nwaf359

**Published:** 2025-08-30

**Authors:** Si Shen, Si Ma, Yong-Qiang Tian, Shun-Li Zhou, Yong-Ling Ruan

**Affiliations:** State Key Laboratory of Maize Bio-breeding, College of Agronomy & Biotechnology, China Agricultural University, China; Beijing Key Laboratory of Growth and Developmental Regulation for Protected Vegetable Crops, College of Horticulture, China Agricultural University, China; Beijing Key Laboratory of Growth and Developmental Regulation for Protected Vegetable Crops, College of Horticulture, China Agricultural University, China; State Key Laboratory of Maize Bio-breeding, College of Agronomy & Biotechnology, China Agricultural University, China; State Key Laboratory for Crop Stress Resistance and High-Efficiency Production and College of Horticulture, Northwest A&F University, China; Division of Plant Sciences, Research School of Biology, The Australian National University, Australia

Increasing crop yield through enhancing photosynthesis has been a long-lasting effort among the plant science community for achieving global food security. However, increasing photosynthesis per se has proven, in many cases, insufficient or even futile to achieve higher yield, indicating a knowledge gap in translating photosynthetic capacity into yield output [1]. A critical step linking source-to-sink partitioning is phloem loading, in which assimilates, mainly as sucrose, are loaded into the phloem of source leaves for translocation to sink organs [2]. In most crops, such as maize, tomato and potato, this process takes place apoplasmically, which consists of two distinctive membrane-transfer steps, namely, SWEET (Sugar Will Eventually Be Exported Transporter)-facilitated passive efflux of sucrose from the phloem parenchyma cells (PPCs) along a concentration gradient and the following SUT/SUC (SUcrose Transporter/Carrier)-mediated active uptake against a concentration gradient by the companion cells (CCs) of the phloem in leaves (Fig. 1a).

**Figure 1.**
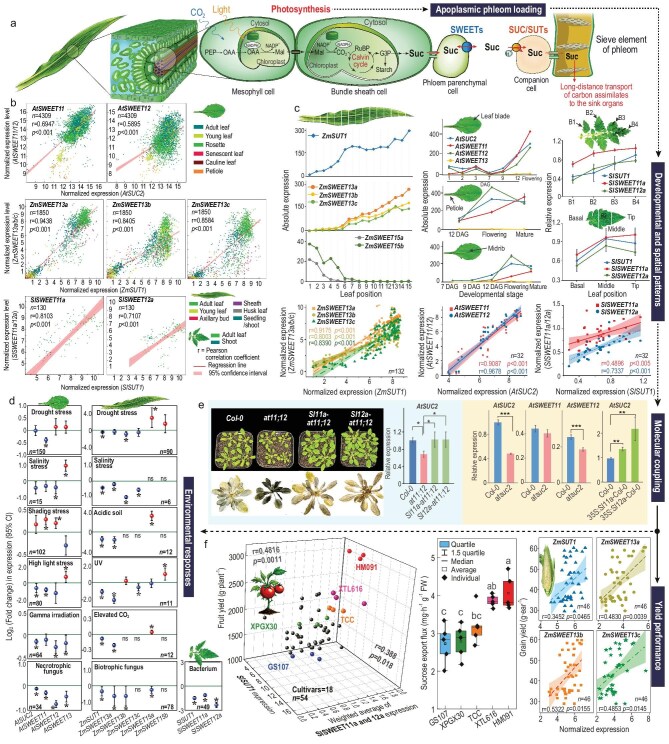
Transcriptional coordination of phloem loading genes, *SUC/SUTs* and *SWEETs*, in development, stress response, and sucrose export capacity for yield formation. (a) A schematic illustration of SUT- and SWEETs-facilitated apoplasmic phloem loading of photosynthetic product, sucrose, using maize leaf as an example. *SWEETs* and *SUC/SUTs* are highly correlated to those genes involved in photosynthesis in maize, tomato and *Arabidopsis* (Table S5). PEP, phosphoenolpyruvate; G3P, glyceraldehyde-3-phosphate; Mal, malate; OAA, oxaloacetate; Suc, sucrose. (b) In different leaf types of *Arabidopsis*, maize and tomato, the *SUC/SUTs* and *SWEETs* facilitating phloem loading are positively correlated. Data was based on 4309, 1850, and 130 leaf samples of *Arabidopsis*, maize and tomato, pooled from 215, 72, and 9 independent studies, respectively. The color of plots demonstrates leaf types. The data source is attached in Table S2. (c) These *SWEETs* and *SUC/SUTs* are spatial-temporally coordinated in *Arabidopsis*, maize and tomato leaves. Note, in maize, *ZmSUT1* is correlated with *ZmSWEET13a/b/c*, but not their paralogs, *ZmSWEET15a/b*. In *Arabidopsis, AtSWEETs* and *AtSUC2* were simultaneously increased and reached the highest level at the flowering stage, exhibiting significant correlation during leaf development. The spatial-temporal information of maize and *Arabidopsis* was extracted from Li *et al*. (2010) and Klepikova *et al*. (2016), respectively, and that of tomato was measured by the current study. (d) *SWEETs* and *SUC/SUTs* are co-responding to environmental stimulus. The fold changes of *SWEETs* and *SUC/SUTs* expressions relative to respective controls in response to specific stresses were integrated by *meta*-analyses with large-scale transcriptomic data from independent studies (Table S2). Drought, soil relative water content (SRWC) was reduced to 30%–66.2%; control, without water limitation with SRWC being held above 80%. Salinity stress, *Arabidopsis* on Skoog medium with 50 mM NaCl, maize with 200 mM NaCl applied to pots; control, without NaCl application. Acidic soil, pH 4.1; control, soil pH 5.5. High light, 700–1300 μmol m^−2^ s^−1^; shading, 39.8–50 μmol m^−2^ s^−1^; control, 50–100 μmol m^−2^ s^−1^. Gamma, 100–200 Gy; control, without gamma irradiation. Elevated CO_2_, 700–750 ppm; control, ambient CO_2_, 350–370 ppm. Bacterium (*Pseudomonas syringae*) and Necrotrophic fungus (*Alternaria Brassicicola* and *Sclerotinia sclerotiorum*) strains were directly applied to the leaves, Biotrophic fungus (*Ustilago Maydis*) suspension was injected into the stem (see Table S7 for details of all treatments). The error bar indicates the 95% confidence interval. * indicates significant difference (*p *< 0.05) between treatments and control. (e) Molecular coupling between *AtSUC2* and *AtSWEET11/12*. Heterologous expressions of *SlSWEET11a* and *12a* restored *AtSUC2* expression and the growth of *atsweet11;12*, and prevented starch accumulation in its leaves at the end of the night (8:00 am). The *atsuc2* displayed a significant reduction in *AtSWEET12* transcript. (f) *SWEETs* and *SUC/SUTs* correlate with sucrose export capacity and yield performance among tomato and maize genotypes. Three-dimensional quadrants according to the levels of *SlSUT1* (x axis), *SlSWEET11a/12a* (weighted average, z axis) and fruit yield (y axis) of 18 commercial tomato cultivars demonstrate that these loading facilitators were significantly correlated with fruit yield. Note that sucrose export flux corresponds to the mRNA levels of loading facilitators and yield (one-way ANOVA). Consistently, in 23 different inbreds of maize, *ZmSUT1* and *ZmSWEET13a/b/c* levels in the source leaf also showed significant correlation with grain yield, whilst the paralogs *ZmSWEET15a/b* were not (Fig. S10, Table S10). Correlation is indicated by Pearson's correlation coefficient with Bonferroni correction to test the statistical significance (as demonstrated by the Bonferroni adjusted *p*-value). Log_2_-normalization was applied to gene expression values.

While the requirement of some individual SWEETs and SUC/SUTs for phloem loading has been demonstrated, it remains unexamined whether and under what conditions these effluxers and influxers are coordinated to load sucrose to the phloem for translocation into sinks such as developing seed and fruit to realize yield potential [2,3]. It is, however, challenging to address this question, in part, due to the close proximity between the PPCs and CCs where SWEETs and SUC/SUTs function, respectively, and also to test this through a single experimental design to cover a wide range of scenarios for a broad appreciation.

To tackle those impediments, we conducted a large-scale *meta*-analysis on transcriptomes from representative apoplasmic loaders, *Arabidopsis*, maize and tomato, covering both monocot and dicot. Several SWEETs or/and SUC/SUTs have been demonstrated to be required for apoplasmic phloem loading in these species, including *AtSUC2* and *AtSWEET11/12* in *Arabidopsis, ZmSUT1* and *ZmSWEET13a/b/c* in maize, and *SlSUT1* in tomato [1, 2, 4,5]. Although the role of SWEETs in phloem loading is yet to be reported in tomato, *SlSWEET11a* and *12a* were phylogenically grouped with *AtSWEET11/12* (Clade III) and highly expressed in leaves (Fig. S1). Indeed, heterologous expressions of *SlSWEET11a* and *12a*, respectively, in *atsweet11;12* double mutant restored starch and growth phenotype (Fig. 1), indicating their roles in phloem loading, hence rendering their inclusion in this study. To improve the reliability of analyses, we chose *AtSWEET13* and *ZmSWEET15a/b*, paralogs of *AtSWEET11/12* and *ZmSWEET13a/b/c*, respectively, but not involved in phloem loading [2,4], as negative controls (Table S1).

Aiming for global insights, we chose the transcriptome datasets covering a wide range of developmental stages and environmental conditions, containing 5674, 2200 and 270 datasets of leaf transcriptome from 273, 95 and 22 studies in *Arabidopsis*, maize and tomato, respectively (Fig. S2; Table S2). A global co-expression analysis with all expressed transcripts identified the top correlated genes to these loading facilitators of *SWEETs* and *SUC/SUTs* (Tables S1 and S3; Fig. S2). It is evident that *AtSUC2, AtSWEET11* and *12* were significantly co-expressed with each other among 23 961 expressed transcripts, where *AtSWEET11* and *12* were ranked the 3rd and 33rd co-expressed genes to *AtSUC2*, with *r* values of 0.7282 and 0.5784, respectively, and *vice versa* (*AtSUC2* ranked the 7th and 10th to *AtSWEET11* and *12*, respectively) (Table S3). Similarly, in maize, *ZmSWEET13a*/*b*/*c* and *ZmSUT1* were the most co-expressed genes with each other (*r* = 0.8405–0.9438) (Fig. S3; Table S3). In tomato, *SlSWEET11a* was also one of the most correlated genes to *SlSUT1* (*r* = 0.8103), with *SlSWEET11a* and *12a* correlated with each other (Table S3).

To understand the expression relationship from a developmental perspective, a co-expression analysis of *SWEETs* and *SUC/SUTs* was conducted on a range of above-ground organs, including different leaf types across developmental stages. Overall, *AtSUC2, ZmSUT1* and *SlSUT1* were significantly positively correlated to *AtSWEET11/12* (*r* = 0.6947 and 0.5895), *ZmSWEET13a/b/c* (*r* = 0.9438, 0.8405 and 0.8584) and *SlSWEET11a/12a* (*r* = 0.8103 and 0.7107), respectively (Fig. 1b). In contrast, the negative controls, *AtSWEET13* and *ZmSWEET15a/b* were not correlated with *AtSUC2* (*r* = 0.3076) and *ZmSUT1* (*r* = −0.1184 and −0.4669), respectively (Fig. S4).

Given that sucrose is the end-product of photosynthesis, we examined the relationship between the transcript levels of the loading facilitators and those for photosynthesis. Zooming into the top 200 genes co-expressed with the loading facilitators among all transcripts (from each of the three species) revealed 56, 140 and 60 genes simultaneously co-expressed with both *SWEETs* and *SUC/SUTs* in *Arabidopsis*, maize and tomato, respectively. Interestingly, within them, 5, 34 and 8 genes were involved in the photosynthetic light reaction processes and/or carbon assimilation in *Arabidopsis*, maize and tomato, respectively (Fig. S5; Tables S4, S5). In addition, several *ZmSUT1*– and *ZmSWEET13a*–co-expressed photosynthesis genes have been selected during crop domestication [6], including those encoding NAD(P)H-quinone oxidoreductase, pyruvate orthophosphate dikinase, ATP-dependent protease subunits, and phosphate transporter protein 5 (Table S6). Consistently, in *Arabidopsis* and tomato, some orthologous genes encoding proteins for photosynthesis were also significantly co-expressed to *SWEETs* and *SUC/SUTs* (Table S5). These results imply a transcriptional association between photosynthesis and apoplasmic phloem loading.

Leaf photosynthetic capacity is gradually established during sink-to-source transition, which prompted us to investigate if *SWEETs* and *SUC/SUTs* were spatially-temporally coupled during leaf development. Results show that the mRNA levels of *ZmSWEET13a/b/c* and *ZmSUT1* progressed from base to tip of the 3rd leaf undergoing sink-to-source transition and significantly correlated with each other, a feature absent for the orthologs, *ZmSWEET15a/b* (Fig. 1c). This spatial pattern matches well with the sink-to-source transition of maize leaves in which the tip region becomes capable for assimilate export first [2]. A similar spatial pattern and correlations were also observed for *SlSWEET11a/12a* with *SlSUT1* in tomato (Fig. 1c). Interestingly, *AtSWEET11/12* exhibited similar rhythmic patterns and significant correlations with *AtSUC2* following exposure to continuous light, while *AtSWEET13* was hardly detectable (Fig. S6). The findings support the hypothesis that the effluxer and influxer genes were simultaneously regulated to execute phloem loading during leaf development.

Aside from the developmental scenarios under normal conditions, the analysis also revealed that the two groups of transporter genes were mostly co-regulated in response to external stimulus (Fig. 1d; Table S7). In response to those underground abiotic stresses, including drought, salinity and acidity, most *SWEETs* and *SUC/SUTs* facilitating apoplasmic loading were significantly down-regulated, except for *ZmSWEET13a, AtSUC2* and *AtSWEET12* under drought and *AtSWEET11* under salinity (Fig. 1d), suggesting the stressed root system may generate and transmit signals to the shoot to repress the phloem loading genes. By contrast, the controls, *AtSWEET13* and *ZmSWEET15a*, were significantly upregulated under salinity for the former and under acidity and drought for the latter (Fig. 1d). Consistently, *AtSWEET11* or *12* and *AtSUC2* were significantly co-repressed by aboveground stress of high light or gamma irradiation, and *ZmSUT1* and *ZmSWEET13a* were also significantly repressed upon UV exposure, with validation by qPCR (Fig. 1d; Fig. S7). The stress-induced downregulation of *SWEETs*-*SUC/SUTs*, however, is not universal since shading treatment upregulated *AtSWEET11* and *12* (Fig. 1d). Notably, in response to elevated CO_2_, *AtSWEET11* and, to a lesser extent, *AtSUC2* were increased in their expressions, whereas *ZmSUT1* and *ZmSWEET13a* were significantly suppressed (Fig. 1d; Fig. S8), indicating distinct responses of C3 and C4 plants. This finding is consistent with a report that elevated CO_2_ increased photosynthesis and biomass of C3 plants but reduced stomatal conductance and transpiration of C4 plants [7].

Altering sugar allocation is an integral component of plant defense against pathogen infection. We thus investigated how the transporter genes for phloem loading may react to biotic stresses. Infection of bacterium, necrotrophic or biotrophic fungi in the leaves of *Arabidopsis*, tomato and maize resulted in significant co-suppression of these *SWEETs* and *SUC/SUTs* (Fig. 1d). Furthermore, these *SWEETs* and *SUTs* exhibited significant co-expression in response to these biotic stresses in their respective species (Fig. S9). This likely blocks sucrose loading to the phloem for export, which could redirect photosynthetic sugars to the infected leaf areas to feed pathogens. It is thus plausible that sustaining the expressions of loading facilitators for sucrose translocation out of the leaves could be a new avenue to tackle pathogen attack.

To assess if the coordinated expression between effluxers and influxers is causally dependent on each other, we exploited relevant *Arabidopsis* mutants. In the *atsuc2* mutant, reduced *AtSUC2* level led to a significant decrease in *AtSWEET12* (Fig. 1e), but without affecting *AtSWEET11*, probably due to some unique features between *AtSWEET11* and *12* promoters (Table S8). Conversely, the *atsweet11;12* double mutant, with sucrose loading being blocked [4], was significantly reduced in *AtSUC2* expression (Fig. 1e). Importantly, heterologous expression of *SlSWEET11a* or *12a* in *Col-*0 and *atsweet11;12* enhanced and recovered the expression of *AtSUC2*, respectively (Fig. 1e). These findings indicate a transcriptional coupling between *SWEETs* and *SUC/SUTs* for phloem loading.

Molecular coupling could be modulated by shared transcriptional networks and signaling pathways. Genes co-expressed with both *SWEETs* and *SUC/SUTs* include those encoding serine/threonine-protein phosphatase, PP2A and transcription factors (TFs) from the DoF and NAC families (Table S4). PP2A is a negative regulator of Snf1-Related Protein Kinase1 (SnRK1), and is required for the transduction of sugar signals to transcriptionally activate many sugar-inducible genes, including *SUC/SUTs* and *SWEETs* [8]. DoF1 can bind the promoters of *AtSUC2* [9], *OsSUT1* and *OsSWEET11* [10], thereby controlling sucrose loading in *Arabidopsis* and rice. The shared DoF-binding *cis*-elements identified in the promoter regions of the *AtSUC2* and *AtSWEET11/12* (Table S8) highlight the possibility that DoF may contribute to their co-expression. Noticeably, *AtSWEET11/12* and *AtSUC2* also shared many other common *cis*-regulatory elements, including hormone-responsive elements (ABRE, CGTCA- and TGACG-motifs for ABA or JA) and several light-responsive elements (CAT-box, G-box, GT1-motif and TCT-motif) (Table S8). These common *cis*-elements are likely targeted by shared TFs to modulate *SWEETs* and *SUC/SUTs* in response to developmental or environmental stimulus. These findings provide clues for future studies to elucidate the molecular mechanism underlying the co-expression of *SWEETs* and *SUC/SUTs* for phloem loading.

The expression levels of *SWEETs* and *SUC/SUTs* varied greatly among maize and tomato genotypes (Fig. 1f), indicating an inherent genotypic difference in the capacity of *SWEETs-* and *SUC/SUTs-*facilitating apoplasmic loading. We then tested whether the expressions of the effluxer and influxer genes matched yield performance in 18 commercial tomato cultivars that varied in fruit yield (Table S9). Among them, HM901 and GS107 showed the highest (3103.8 g·plant^−1^) and lowest yield (1172.9 g·plant^−1^), with the highest (4.06 mg·h^−1^·g^−1^ FW) and lowest (2.73 mg·h^−1^·g^−1^ FW) sucrose efflux capacity, respectively (Fig. 1f; Table S9). Notably, the levels of *SlSWEET11a/12a* and *SlSUT1* in mature leaves corresponded to the sucrose efflux capacity and were significantly correlated with the final yield performance among cultivars (Fig. 1f). Similarly, in 23 distinct maize inbreeds, the levels of *ZmSUT1* and *ZmSWEET13a/b/c* were positively correlated with the final grain yield (Fig. 1f; Table S10), a phenomenon absent for the negative controls, *ZmSWEET15a* and *15b* (Fig. S10). The data suggest that the *SWEETs* and *SUC/SUTs* transcription nexus is closely associated with sucrose export capacity and yield performance of tomato and maize cultivars, and therefore could be a potential target in molecular breeding for realizing yield potential.

Taken together, our findings demonstrate a close transcriptional coupling between the sucrose effluxer and influxer genes for phloem loading in development, environmental responses and yield formation across different species, opening new avenues for optimizing resource allocation to improve crop yield.

## Supplementary Material

nwaf359_Supplemental_Files

## References

[1] Ruan Y-L . Annu Rev Plant Biol 2014; 65: 33–67. 10.1146/annurev-arplant-050213-040251 24579990

[2] Braun DM . Annu Rev Plant Biol 2022; 73: 553–84. 10.1146/annurev-arplant-070721-083240 35171647

[3] Shen S, Ma S, Wu L et al. Trends Plant Sci 2023; 28: 893–901. 10.1016/j.tplants.2023.03.015 37080837

[4] Chen L-Q, Qu X-Q, Hou B-H et al. Science 2012; 335: 207–11. 10.1126/science.1213351 22157085

[5] Hackel A, Schauer N, Carrari F et al. Plant J 2006; 45: 180–92. 10.1111/j.1365-313X.2005.02572.x 16367963

[6] Hufford MB, Xu X, van Heerwaarden J et al. Nat Genet 2012; 44: 808–11. 10.1038/ng.2309 22660546 PMC5531767

[7] Ainsworth EA, Long SP. New Phytol 2005; 165: 351–72. 10.1111/j.1469-8137.2004.01224.x 15720649

[8] Li Z, Wei X, Tong X et al. Mol Plant 2022; 15: 706–22. 10.1016/j.molp.2022.01.016 35093592

[9] Schneidereit A, Imlau A, Sauer N. Planta 2008; 228: 651–62. 10.1007/s00425-008-0767-4 18551303

[10] Wu Y, Lee S-K, Yoo Y et al. Mol Plant 2018; 11: 833–45. 10.1016/j.molp.2018.04.002 29656028

