# Supplementary material for: Synchronizing sucrose effluxers with influxers in phloem loading for yield output and adaptation to environment
Source: Natl Sci Rev. 2025 Aug 30;12(10):nwaf359. doi: 10.1093/nsr/nwaf359 (PMC12492195; doi:10.1093/nsr/nwaf359)
Supplement: nwaf359_Supplemental_Files [file nwaf359_supplemental_files.zip › Supplementary Materials_R3.docx]

**Supplementary Materials for**

**Synchronizing Sucrose Effluxers with Influxers in Phloem Loading for Yield Output and Adaptation to Environment**

Si Shen^1#^, Si Ma^2#^, Yong-Qiang Tian^2^, Shun-Li Zhou^1^ and Yong-Ling Ruan^3,4^*

^1^State Key Laboratory of Maize Bio-breeding, China Agricultural University, Beijing 100193, China.

^2^Beijing Key Laboratory of Growth and Developmental Regulation for Protected Vegetable Crops, College of Horticulture, China Agricultural University, Beijing 100193, China

^3^State Key Laboratory for Crop Stress Resistance and High-Efficiency Production and College of Horticulture, Northwest A&F University, Xianyang, 712100, China

^4^Division of Plant Sciences, Research School of Biology, The Australian National University, Canberra, ACT, 2601 Australia

^#^These authors contributed equally to the work

^*^For correspondence (e-mail:) yong-ling.ruan@anu.edu.au

**This** **file includes:**

Supplementary Text S1

Supplementary Figures S1 to S11

References cited by supplementary materials

**Supplementary Text S1**

**Materials and methods**

**Plant materials and growth conditions**

Tomato (*Solanum lycopersicum*, cultivar of Ailsa Craig) and Arabidopsis (*Arabidopsis thaliana*) were used as the wild type (WT) to verify the expression patterns of *SWEETs* and *SUC/SUTs* in different leaf positions and in response to diurnal, circadian and light strengthening. *Arabidopsis* mutants, *atsweet11;12* (Salk_073269 and Salk_031696 T-DNA insertions) (Chen *et al.*, 2012) and *atsuc2* (Salk_208617C T-DNA insertion), were obtained from the Arabidopsis Biological Resource Center. Transgenic lines of *Arabidopsis* were generated in this study by heterologous expressions of *SlSWEET11a* and *12a* in *Col-0* and *atsweet11;12* double mutants. The genomic sequences of *SlSWEET11a* (903 bp of the entire coding region) and *SlSWEET12a* (897 bp of the entire coding region) were cloned and ligated into the *pCAMBIA1300-NOS* between *Hind*III and *Xba*I. The resultant plasmids were confirmed by sequencing and transformed into the *atsweet11;12* mutant using the *Agrobacterium*-mediated floral dip method (Zhang *et al.*, 2006).

Tomato plants were grown in a greenhouse under natural light with day and night temperatures of 25 and 18 °C for 14 and 10 h, respectively. Plants were raised in pots (10 cm×10 cm) filled with potting mix (part coarse sand: part perlite: part coir-peat=1:1:1) and slow-released fertilizer as previously described (Ru *et al.*, 2017). In most cases, *Arabidopsis* plants were grown in pots with potting mix in an incubator with 12 h day (22°C) and 12 h night (18°C). For the *in vivo* starch staining of mutant resettles, 25-day-old *Arabidopsis* plants were incubated in prolonged dark for 40 h prior to starch staining. For light intensity treatments, *Arabidopsis* were raised in an incubator under a long photoperiod (16 h light, 22°C; 8 h dark, 18°C) with an ambient light (200 µmol m^-2^ s^-1^). Then, 28-day-old seedlings were transferred into incubators with shading (50 µmol m^-2^ s^-1^) or high light (800 µmol m^-2^ s^-1^) conditions, respectively.

**Transcriptome datasets and co-expression analysis**

All transcriptomic data generated by RNA-seq and Micro-array were collected from Gene Expression Omnibus (Edgar *et al.*, 2002) and ArrayExpress (Parkinson *et al.*, 2007), respectively, except for the spatial transcriptome on maize leaf that was obtained from the Maize eFP Browser at http://bar.utoronto.ca. Only transcriptomic data on leaves and those photosynthetic tissues (husk, seedling, sheath, shoot) were included in subsequent analyses. Data was selected from studies which provided comparative information for gene expressions in different genotypes, positions and developmental stages of leaves, or under various conditions including biotic stresses and abiotic stresses (Table S7). Data source and sample details of these involved transcriptomes were listed in Table S2. To compare the expression values across samples, treatments and experiments, the raw data generated by micro-array was processed using quantile normalization with the algorithms from the Bioconductor limma package and global scaling (Gentleman *et al.*, 2004; Ritchie *et al.*, 2015). For RNA-seq dataset, these adapters from the reads were removed using Trimmomatic (Bolger *et al.*, 2014). The expression values were estimated in terms of transcript per million (TPM) via mapping libraries of reads to transcriptome references (Langmead *et al.*, 2009; Li & Dewey, 2011). The absolute expression values for *SWEETs* and *SUC/SUTs* were extracted from each biological replicate as previously described (Xu *et al.*, 2018). The targeted genes (and corresponding gene IDs) encoding SWEETs and SUC/SUTs in apoplasmic loading were listed in Table S1.

The correlation-based co-expression network analyses were based on the RNA-seq datasets in maize and tomato, as higher accuracy of RNA-seq in transcript profiles (Hruz *et al.*, 2008), but based on micro-array datasets, the majority data type, in *Arabidopsis* (Fig. S4, Table S2). Identification of the candidates and networks closely correlated to *SWEETs* and *SUC/SUTs* was done by visualizing co-expression relationships beyond the threshold (Pearson’s correlation coefficient with the querying transporter genes) and circular clustering via the Co-Expression tool in Genvestigator (Nebion AG, Hruz *et al.*, 2008). This coefficient was calculated from log_2_-nomalized TPM values of gene expression. A broader correlated networks among these top correlated candidates were indicated by connecting lines with the thresholds in *Arabidopsis* (Pearson’s coefficient> 0.6), maize and tomato (Pearson’s coefficient> 0.9). The requirement for distinct expression variation thresholds across species arose because maize and tomato exhibited markedly elevated coefficients in gene expression compared to *Arabidopsis*, probably due to inherently transcriptional heterogeneity in these species. The statistical significance of Pearson’s correlation was indicated by *p* value with Bonferroni Correction.

**Transcriptome meta-analysis**

Transcriptome meta-analysis was performed to evaluate the responses of *SWEETs* and *SUC/SUTs* to external stimulus by using Comprehensive Meta-Analysis (CMA) V3 software according to the CMA manual (https://www.meta-analysis.com/). Briefly, a random-effect model was used. The scaled fold changes (*log_2_FC*) were chosen as the effect size and calculated using Eq (1) (Hedges *et al.*, 1999):

${log}_{2}FC={log}_{2}(\bar{X_{t}}\div\bar{X_{c}})$

Where $\bar{X_{c}}$ and $\bar{X_{t}}$ are the mean values of gene expressions under controlled condition and treatments of external stimulus, respectively. Specifically, the criteria to classify stresses in our transcriptomic meta-analysis included abiotic (drought, salinity, shading, high light, acidic soil, UV, gamma irradiation, and elevated CO_2_) and biotic stresses (infection by necrotrophic fungus, biotrophic fungus, and bacterium) (Fig. 1d, Table S7). The experimental conditions for the treatments were detailed in Table S7, according to the description by the original studies. Variance (*v*) of each *log_2_FC* was calculated using Eq (2):

$$v=\frac{{SD}_{t}^{2}}{n_{t}\bar{\bar{X}_{t}^{2}}}+\frac{{SD}_{C}^{2}}{n_{c}\bar{\bar{X}_{c}^{2}}}$$

where *SD* and *n* are standard deviation and sample sizes, respectively.

The significance of the overall effect was calculated by CMA software and indicated by a 95% confidence interval. A change was considered a significant increase (${log}_{2}FC>0$) or decrease (${log}_{2}FC<0)$ in response to a stimulus at the level of *p* <0.05 when the 95% confidence interval did not overlap with the zero line (Fig. 1d).

**RNA extraction, reverse transcription and qRT-PCR**

Total RNA was isolated from 100 to 200 mg of fresh leaf tissues using Trizol reagent (Invitrogen) according to the manufacturer’s protocol. cDNA was synthesized from 1000 ng of RNA using Super-Script III Reverse Transcriptase (Invitrogen) with oligo (dT)_18_ and random primers. qRT-PCR was performed in triplicate using undiluted and 1:5 diluted cDNA as templates, and the reaction was performed using SYBR Premix Ex TaqTM (Tli RNaseH Plus) as previously reported (Ma *et al.*, 2019). Measurement was on a QuantStudio 6 Flex System (ThermoFisher). Relative expression was calculated as previously described (Pfaffl, 2001). The gene-specific primers were designed using Primer-BLAST (https://www.ncbi.nlm.nih.gov/tools/primer-blast/) and were listed in Table S11.

***In vivo* starch staining**

*In vivo* starch staining of *Arabidopsis* plants was performed according to Chen *et al.*, (2012). Briefly, rosette leaves were harvested from plants grown at 16 h light and 8 h dark cycle, followed by exposure to continuous dark for 40 h. The harvested rosettes were immediately emerged in 80% (v/v) ethanol with 5% (v/v) formic acid at room temperature, then stained with KI_2_ Lugol’s iodine solution and washed in water.

***Sucrose efflux capacity***

The sucrose efflux capacity was quantified by measuring the sucrose content in phloem saps, according to Xu et al. (2018), with slight modifications. Specifically, at 10:00 a.m., two leaflets of tomato were cut from the rachis while keeping the incision emerging in 20 mM EDTA, and then inserted into a 5 mL tube containing about 4 mL EDTA solution (20 mM) under dark condition for 60 min. Afterwards, the leaflets were washed with distilled water to remove EDTA, recut at the base and immediately transferred into another 5 mL tube containing distilled water under natural light and 60%-70% humidity conditions for 5 h to collect the phloem sap. The sucrose content in the phloem sap was measured using a sucrose kit (Solarbio, BC2465) following the kit instructions.

**Correlation analysis with yield**

For the correlation analysis between the expression levels of the *SWEETs* and *SUC/SUTs* and yield outputs among different maize inbreds, the transcriptomic data was extracted from Hufford *et al*., (2021), with that of yield performance extracted from Yang et *al*., (2014) as detained in Table S10. The two studies investigated 23 common maize inbreds with diverse genomic and phenotypic features. Eighteen commercial cultivars of tomato (*Solanum lycopersicum*) were planted in a greenhouse under normal production conditions in the current study. The 15^th^-18^th^ mature leaves of each cultivar were collected at 10:00 am to analyze the levels of *SlSUT1*, *SlSWEET11a* and *12a* by qRT-PCR as described above. Tomato yield was measured by weighing 7 to 8 trusses of mature fruits from each four-month-old plant.

**Statistical analysis**

The measurements were performed with three to eight independent biological replicates as indicated in the captions. The statistical analyses of correlation were indicated by *Pearson* correlation analyses with Bonferroni Correction in Origin 2021. Log_2_-normalization was applied to gene expression in correlation analysis to stabilize variance and to ensure parametric test assumptions are met and to minimize bias from highly expressed genes. Analyses of qPCR were conducted by Student’s *t*-test in Microsoft Excel 2016. Figures were prepared by Microsoft PowerPoint 2016 and Adobe Illustrator CS6.

**Supplementary Figures and References Cited by Captions**


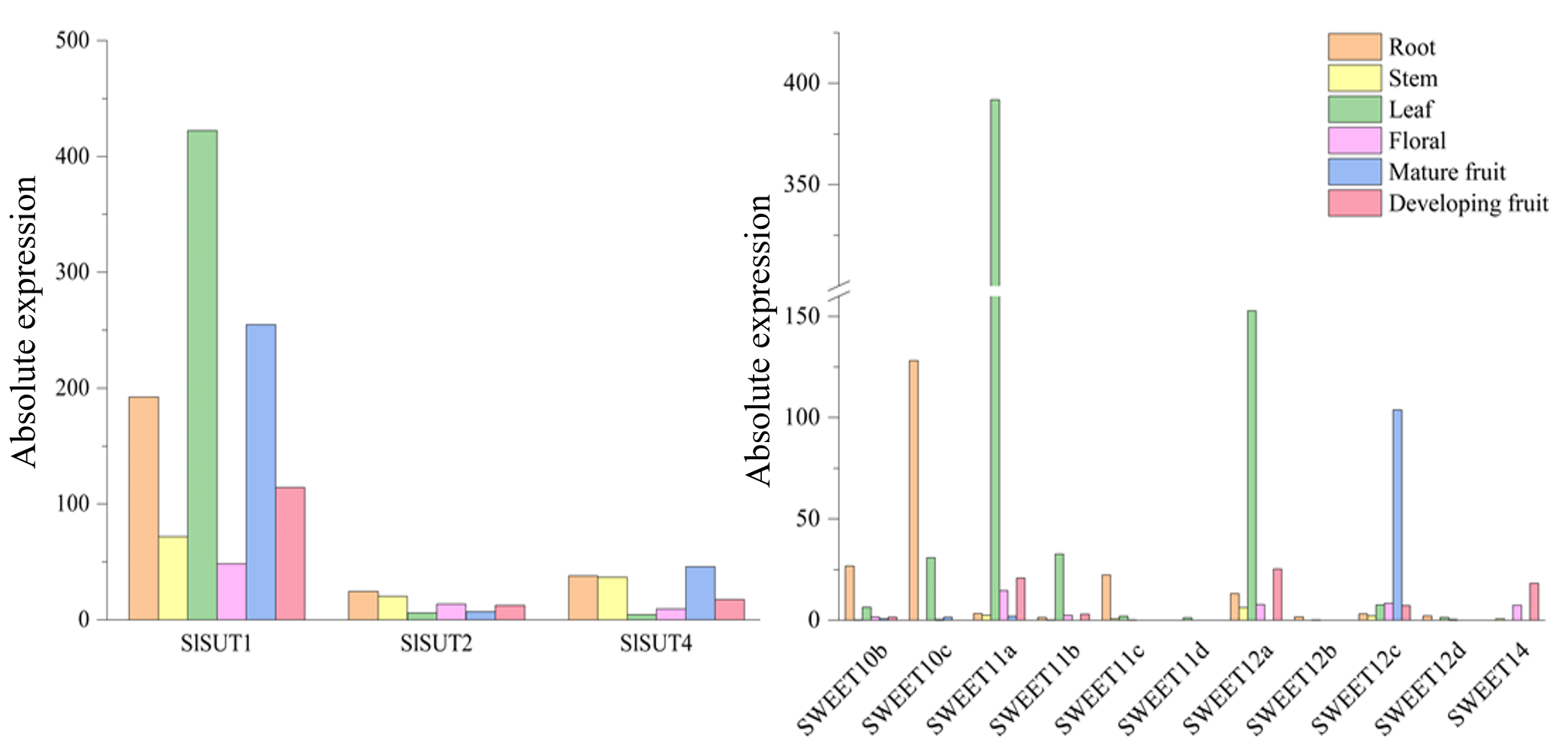


**Supplementary Figure S1. Tissue-specific expressions of *SlSWEETs* and *SlSUTs* families in different organs of tomato.** Note, *SlSWEET11a* and *12a* from the Clade III SWEETs were highly expressed in leaves. Data was extracted from the Tomato Genome Consortium (2012).


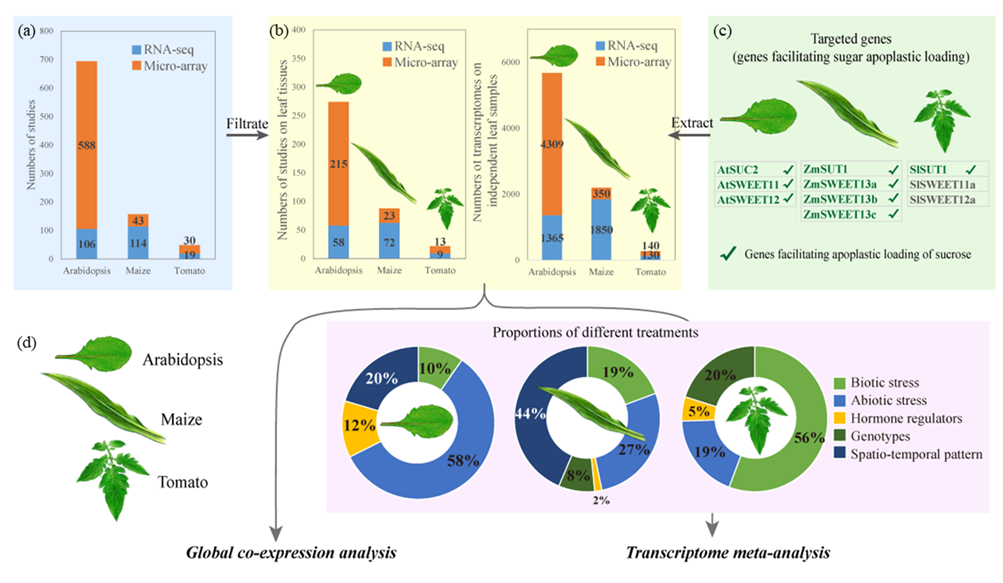


**Supplementary Figure S2. Overview of large-scale analyses on leaf transcriptomes in *Arabidopsis*, maize and tomato.** The numbers of independent studies were included in the transcriptomic dataset of *Arabidopsis*, maize and tomato. All transcriptomes (RNA sequencing or micro-array) were extracted from the database of Genevestigator (https://genevestigator.com/).

a. Numbers and proportions of RNA-seq and Micro-array studies in the dataset.

b. These transcriptomes on leaves or seedlings were filtered by omitting those from other tissues for subsequent analyses.

c. Candidate genes of *SWEETs* and *SUTs* that have been experimentally verified or predicted to facilitate apoplasmic phloem loading of sugar in *Arabidopsis*, maize and tomato.

d. The transcriptomic data with developmental or environmental stimulus were dissected for subsequent meta-analyzing the conditionality of the coordination of *SWEETs* and *SUTs*. All transcriptomes on leaves were included in co-expression analyses.


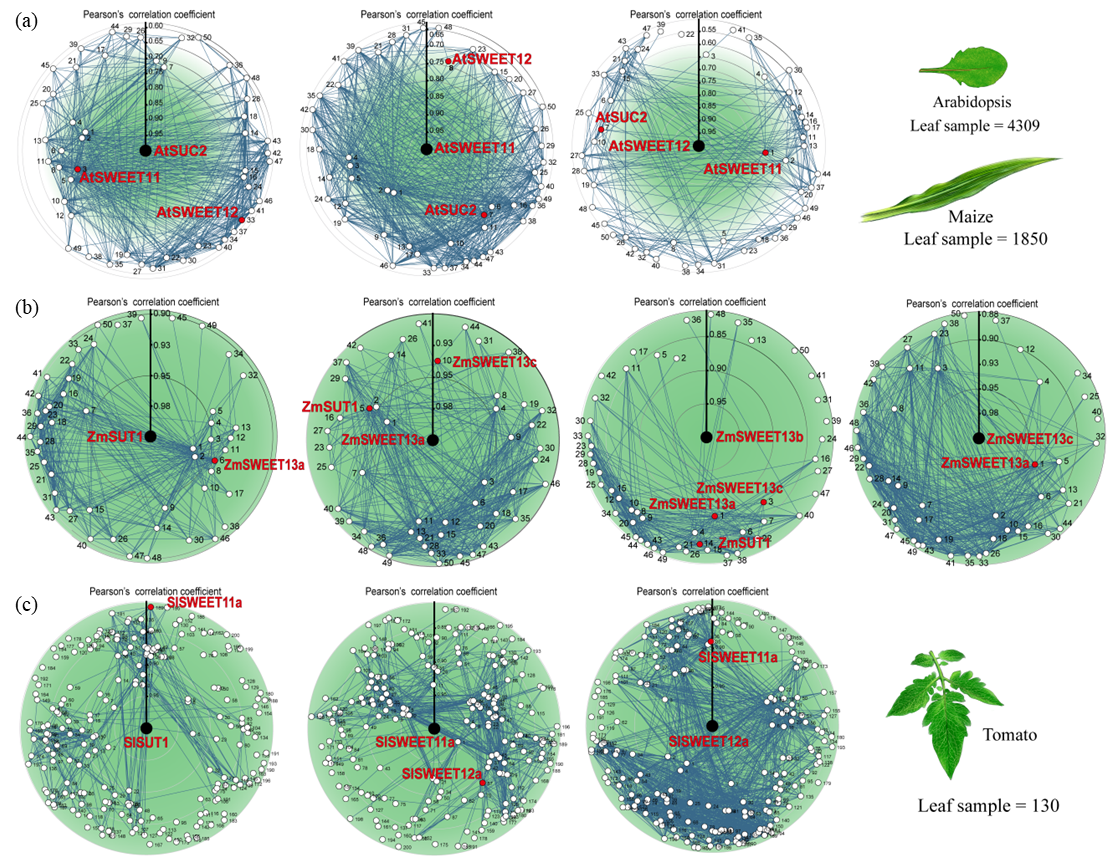
**Supplementary Figure S3. Top co-expressed genes of *SWEETs* and *SUC/SUTs* in transcriptomes on the leaves of *Arabidopsis*, maize and tomato.**

a. Top 50 co-expressed genes to *AtSUC2*, *AtSWEET11* and *AtSWEET12* from 4309 micro-array samples (involved ~23961 genes) on leaf samples. Those with a mutual correlation coefficient above 0.6 (*p* <0.05) were presented with connecting lines.

b. Top 50 co-expressed genes to *ZmSUT1*, *ZmSWEET13a*, *13b* and *13c* from 1850 RNA-sequencing samples (involved over 32000 genes) on leaf samples. Those involved genes with a mutual correlation coefficient above 0.9 (*p*<0.05) were presented with connecting lines.

c. Top 200 co-expressed genes to *SlSUT1*, *SlSWEET11a* and *SlSWEET12a* in the 130 RNA-sequencing samples (involved ~31760 genes) on leaf samples. Those involved genes with mutual correlation above 0.9 (*p*<0.05) were presented with connecting lines.

Analyses were based on *Pearson*’s correlation between the targeted genes (filled black cycle) with all other genes (white cycles indicated those top co-expressed genes). In *Arabidopsis*, maize and tomato, *AtSWEET11*, *ZmSWEET13a* and *SlSWEET11a* were highly co-expressed with *AtSUC2* (r=0.77, ranked 3^rd^), *ZmSUT1* (*r*=0.94, ranked 6^th^) and *SlSUT1* (*r*=0.89, ranked 189^th^), respectively.


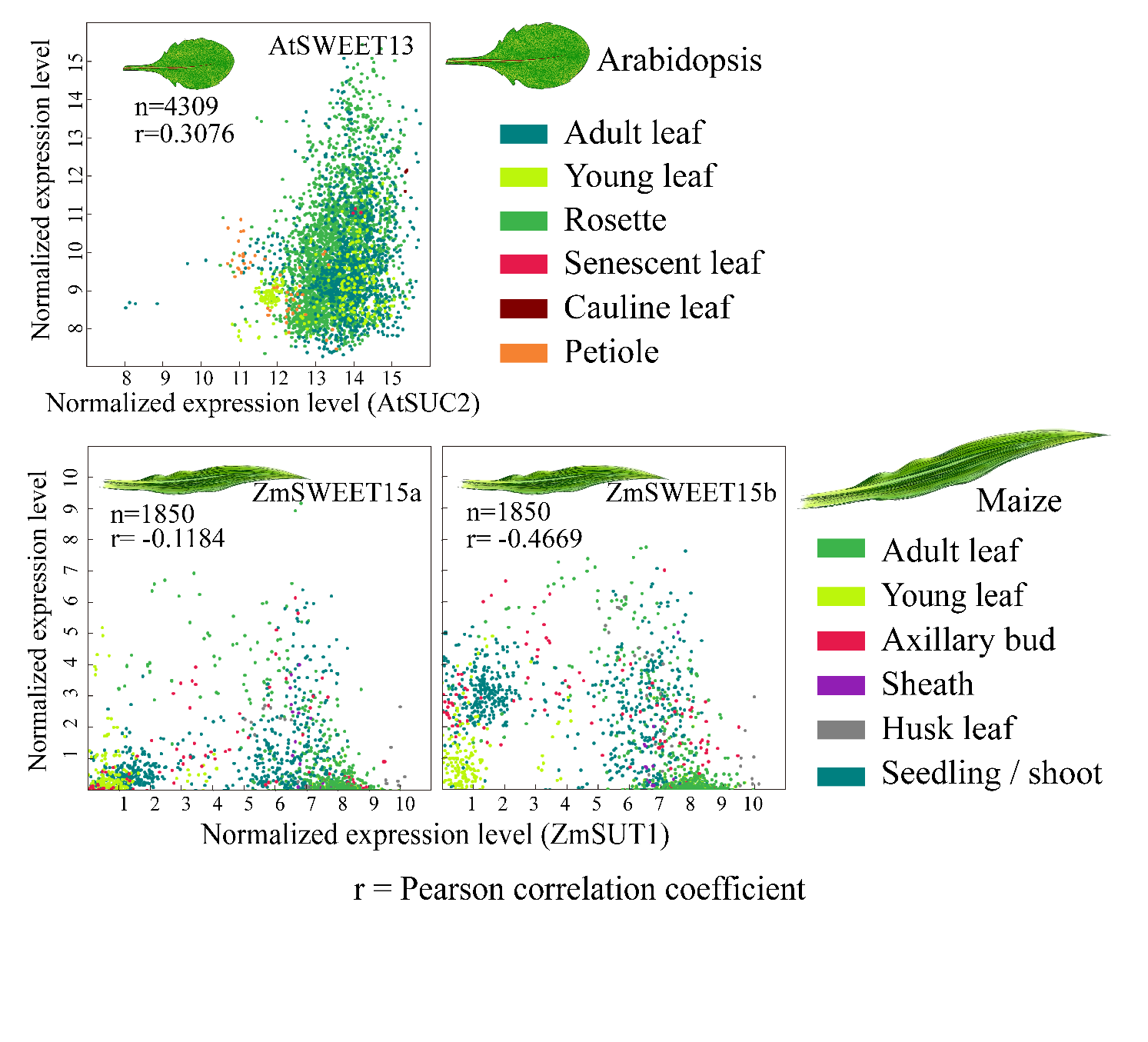


**Supplementary Figure S4. Normalized expression of *AtSUC2* and *ZmSUT1* did not correlate with that of *AtSWEET13* and *ZmSWEET15a/b*, respectively.**

r=Pearson’s coefficient; n=genotype number.


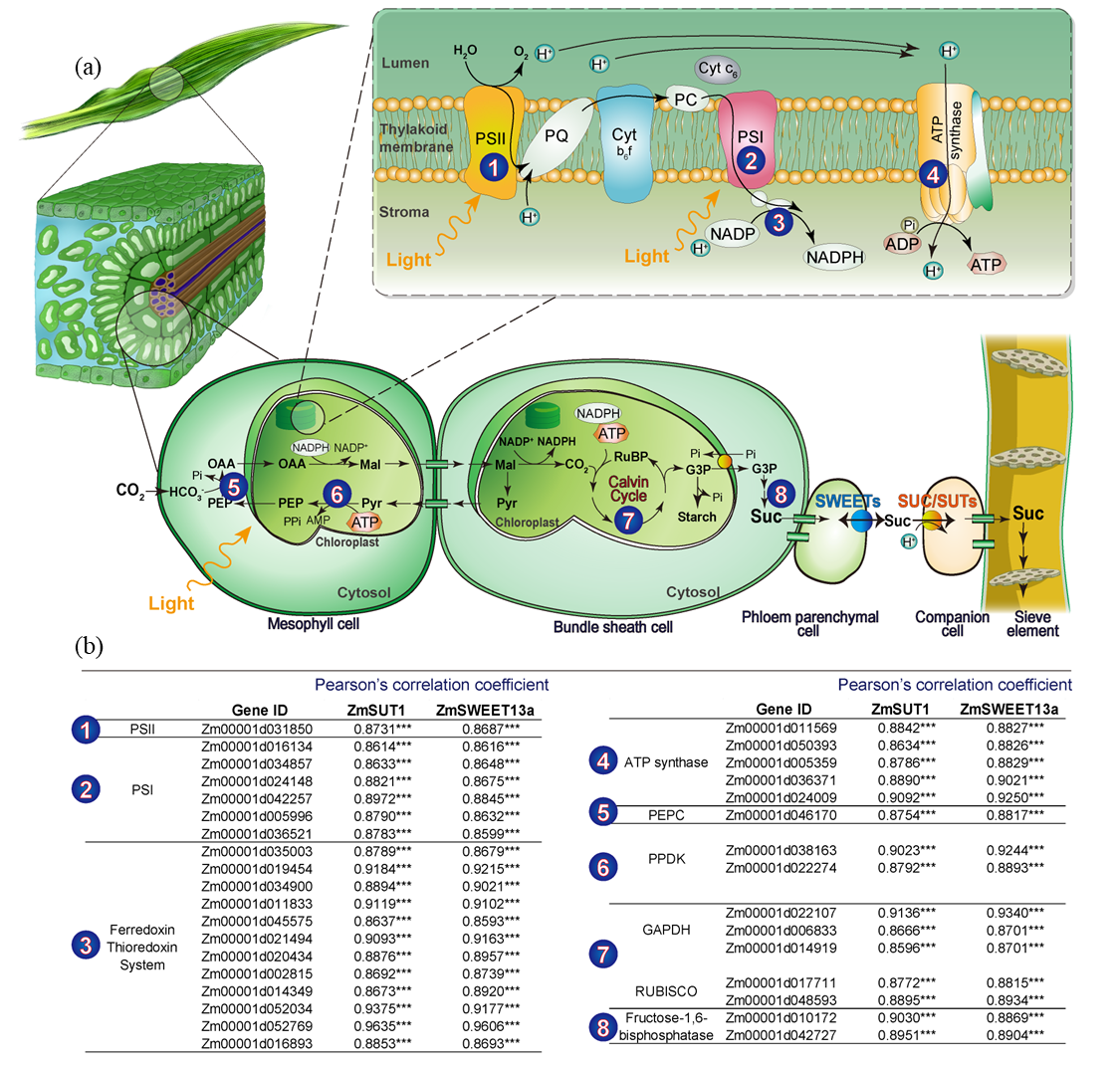


**Supplementary Figure S5. *SWEETs* and *SUC/SUTs* highly correlate to those involved in both light and dark reactions of photosynthesis.**

a. A schematic illustration of C4 photosynthesis using a maize leaf as an example. Here, powered by light, the photosynthesis systems I and II produce NADPH and ATP on the thylakoid membrane, which is used for subsequent fixation of CO_2_ in the chloroplast stroma in the mesophyll cell and then in the bundle sheath cell. Ultimately, sucrose is synthesized in the cytosol of the bundle sheath cell for loading into the phloem (sieve element-companion cell complex) for long-distance transport to sinks or for temporary storage in the chloroplast as starch. In *Arabidopsis* and tomato leaves, the Calvin Cycle and carbon assimilation take place in the mesophyll cells only. G3P, glyceraldehyde-3-phosphate; Mal, malate; OAA, oxaloacetate; PEP, phosphoenolpyruvate; PQ, plastoquinone; Pyr, pyruvate; Suc, sucrose.

b. Genes involved in the light and dark reactions of photosynthesis displayed top correlations simultaneously with *ZmSWEET13a* and *ZmSUT1*. Similar correlations were also observed in *Arabidopsis* or tomato (see Table S5). PS, photosynthesis system; PEPC, PEP carboxylase; PPDK, pyruvate phosphate dikinase; GAPDH, G-3-P dehydrogenase.

*** represents significance at the level of *p*<0.001.


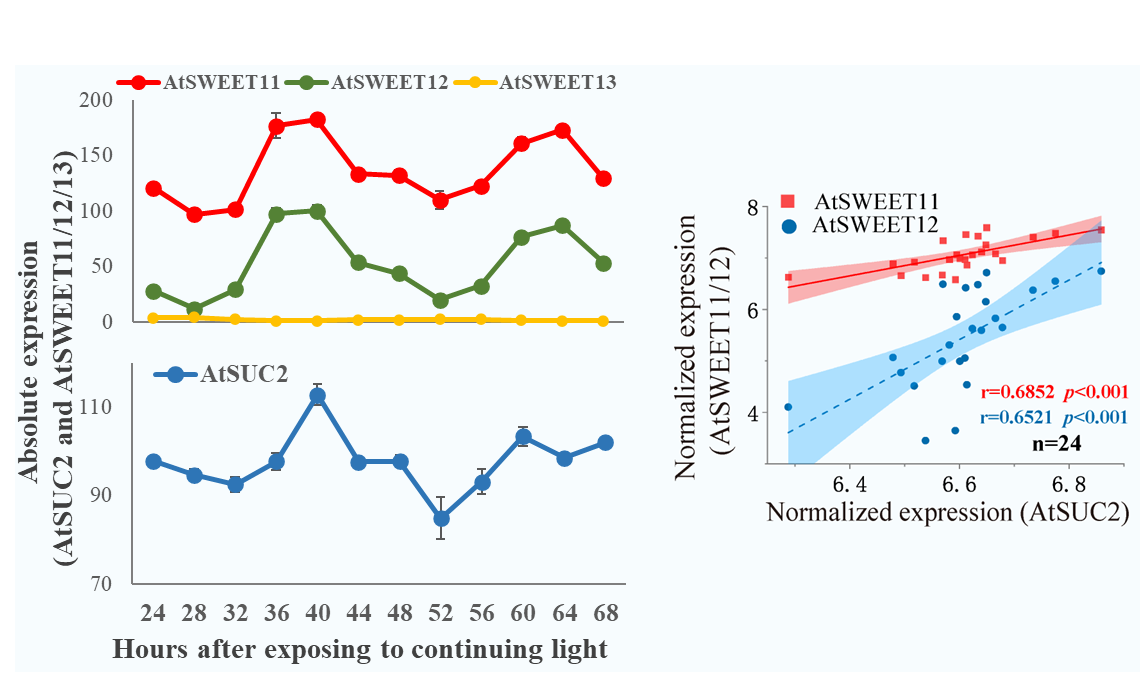


**Supplementary Figure S6.** ***AtSUC2* and *AtSWEET11/12* exhibited significant correlation following exposure to continuous light.**

The RNA-seq data was obtained from rosettes of *Arabidopsis* plants grown under continuous light (50 µmol photons m^-2^ s^-1^) at the specified time intervals, after transferring from a normal growth condition of 12 h-light (50 µmol photons m^-2^ s^-1^) / 12 h-dark cycles at 22°C.


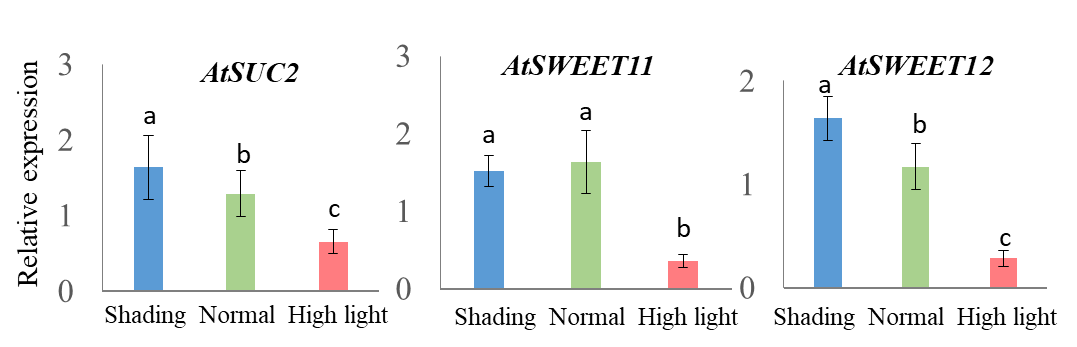


**Supplementary Figure S7. Experimental validation of relative expressions of *AtSUC2*, *AtSWEET11* and *12* in response to shading and high light stresses.**

The expressions of *AtSUC2* and *AtSWEET11/12* were simultaneously suppressed and promoted by exposing to high light (800 µmol m^-2^ s^-1^) and shading (50 µmol m^-2^ s^-1^), respectively, compared with the normal light condition (100 µmol m^-2^ s^-1^), except for *AtSWEET11,* which was not promoted by shading.

One-way ANOVA was determined with Duncan's new multiple range test, n=8, letters indicate the significant difference at *p*=0.05.


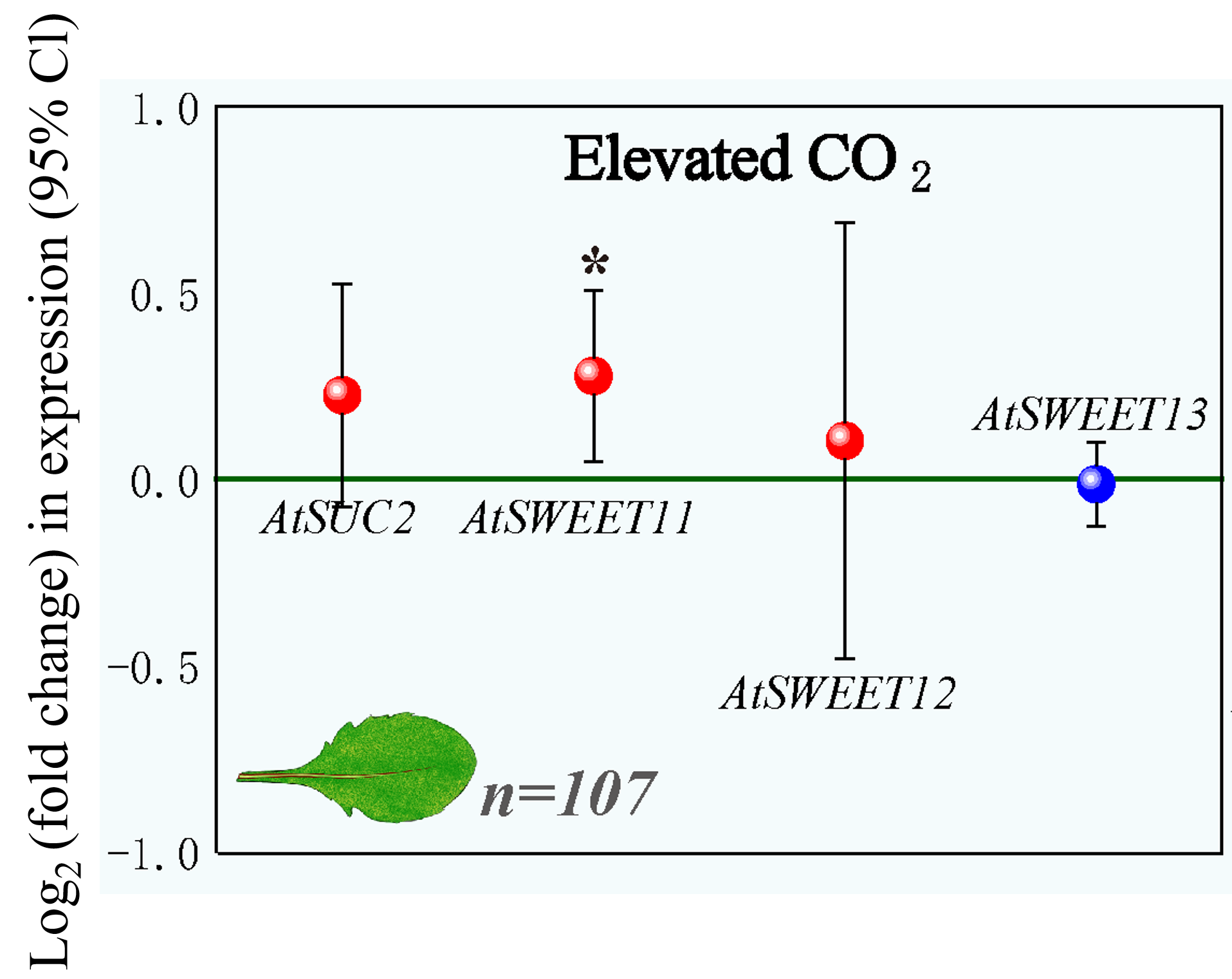


**Supplementary Figure S8. Responses of** ***AtSWEET11/12/13* and *AtSUC2* to elevated CO_2_.**

The fold change of *AtSWEET11/12/13* and *AtSUC2* expressions relative to respective controls in response to the specified stresses were integrated by meta-analyses.

Elevated CO_2_, 700~750 ppm CO_2_, control, ambient CO_2_, 350~370 ppm CO_2_. The error bar indicates the 95% confidence interval (CI) and means. * indicates the significance (*p*<0.05) between stress treatments and control.


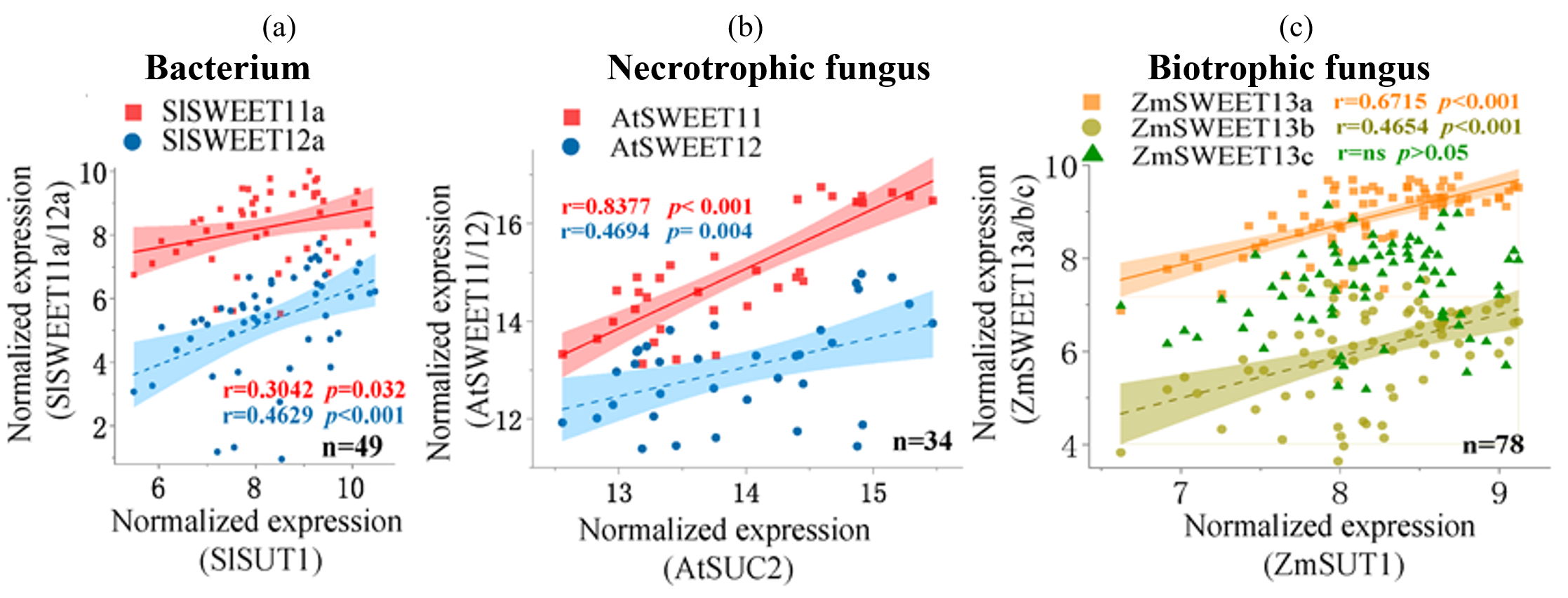


**Supplementary Figure S9. Coordinated expressions of *SWEETs* and *SUC/SUTs* in response to biotic stresses.**

a. Correlation between SWEETs and SUC/SUTs in tomato leaves upon bacterial infection. Bacterium (*Pseudomonas syringae*) was directly applied by dipping the leaf into the bacterial suspension for 2-3 sec. Data was extracted from Rosli *et al*. (2013) and Yang *et al*. (2015).

b. Correlation between SWEETs and SUC/SUTs in Arabidopsis leaves upon necrotrophic fungal infection. Necrotrophic fungus (*Alternaria Brassicicola* and *Sclerotinia sclerotiorum*) droplets were directly applied to the leaves. Data was extracted from Bethke *et al*., (2014) and Henrik U. Stotz *et al.*, (2011).

c. Correlation between SWEETs and SUC/SUTs in maize leaves upon biotrophic fungal infection. Biotrophic fungus (*Ustilago Maydis*) cell suspension was injected into the stem. Data was extracted from Horst *et al*., (2010) and Tanaka *et al.*, (2014).

*r*=*Pearson's* coefficient; *p* < 0.05 indicates significant correlation; n=sample number, the shading indicates 95% confidence interval.


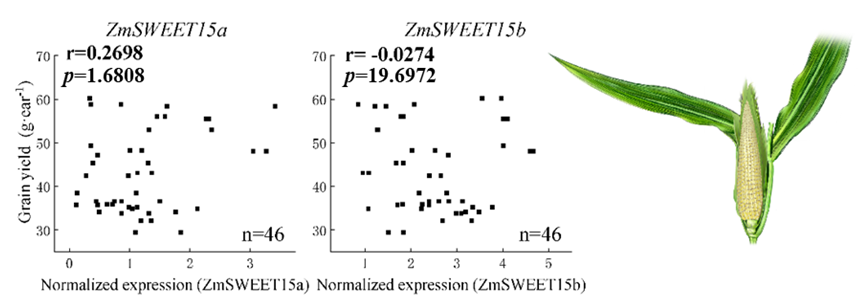


**Supplementary Figure S10. The expressions of *SWEET15a* and *15b* did not show association with grain yield.**

The expressions of *ZmSUT1* and *ZmSWEET13a/b/c* in different maize genotypes were associated with their grain yield performance, but that of *ZmSWEET15a* and *15b* was not (indicated by the Bonferroni-adjusted *p* values). The yield performances and gene expressions of 25 maize genotypes were obtained from Yang *et al*. (2014) and Hufford *et al*. (2021).

**References Cited by Supplementary Materials**

**Bethke G, Grundman RE, Sreekanta S, Truman W, Katagiri F, Glazebrook J. 2014.** *Arabidopsis* PECTIN METHYLESTERASEs contribute to immunity against *Pseudomonas syringae*. *Plant Physiology* **164:** 1093-1107

**Bolger AM, Lohse M, Usadel B. 2014.** Trimmomatic: a flexible trimmer for Illumina sequence data. *Bioinformatics* **30**(15): 2114-2120.

**Chen L-Q, Qu X-Q, Hou B-H, Sosso D, Osorio S, Fernie AR, Frommer WB. 2012.** Sucrose efflux mediated by SWEET proteins as a key step for phloem transport. *Science* **335**(6065): 207-211.

**Edgar R, Domrachev M, Lash AE. 2002.** Gene Expression Omnibus: NCBI gene expression and hybridization array data repository. *Nucleic Acids Research* **30**(1): 207-210.

**Gentleman RC, Carey VJ, Bates DM, Bolstad B, Dettling M, Dudoit S, Ellis B, Gautier L, Ge Y, Gentry J, et al. 2004.** Bioconductor: open software development for computational biology and bioinformatics. *Genome Biology* **5**(10): R80.

**Hedges LV, Gurevitch J, Curtis PS. 1999.** The meta-analysis of response ratios in experimental ecology. *Ecology* **80**(4): 1150-1156.

**Horst RJ, Doehlemann G, Wahl R, Hofmann J, Schmiedl A, Kahmann R, Kaݶmper J, Sonnewald U, Voll LM. 2010.** *Ustilago maydis* infection strongly alters organic nitrogen allocation in maize and stimulates productivity of systemic source leaves. *Plant Physiology* **152:** 293-308.

**Hruz T, Laule O, Szabo G, Wessendorp F, Bleuler S, Oertle L, Widmayer P, Gruissem W, Zimmermann P. 2008.** Genevestigator v3: a reference expression database for the meta-analysis of transcriptomes. *Advances in Bioinformatics* **420747**(5):**2008**.

**Hufford MB, Seetharam AS, Woodhouse MR, Chougule KM, Ou S, Liu J, Ricci WA, Guo T, Olson A, Qiu Y. 2021.** *De novo* assembly, annotation, and comparative analysis of 26 diverse maize genomes. *Science* **373:** 655-662.

**Klepikova AV, Kasianov AS, Gerasimov ES, Logacheva MD, Penin AA. 2016.** A high resolution map of the *Arabidopsis thaliana* developmental transcriptome based on RNA-seq profiling. *The Plant* *Journal* **88:** 1058-1070.

**Lai X, Bendix C, Yan L, Zhang Y, Schnable JC, Harmon FG. 2020.** Interspecific analysis of diurnal gene regulation in panicoid grasses identifies known and novel regulatory motifs. *BMC Genomics* **21:** 1-17.

**Langmead B, Trapnell C, Pop M, Salzberg SL. 2009.** Ultrafast and memory-efficient alignment of short DNA sequences to the human genome. *Genome Biology* **10**(3): R25.

**Li B, Dewey CN. 2011.** RSEM: accurate transcript quantification from RNA-Seq data with or without a reference genome. *BMC Bioinformatics* **12**(1): 323.

**Li P, Ponnala L, Gandotra N, Wang L, Si Y, Tausta SL, Kebrom TH, Provart N, Patel R, Myers CR. 2010.** The developmental dynamics of the maize leaf transcriptome. *Nature Genetics* **42:** 1060.

**Ma S, Sun L, Sui X, Li Y, Chang Y, Fan J, Zhang Z. 2019.** Phloem loading in cucumber: combined symplastic and apoplastic strategies. *The Plant Journal* **90**(3): 391-404.

**Romanowski A, Schlaen RG, Perez‐Santangelo S, Mancini E, Yanovsky MJ. 2020.** Global transcriptome analysis reveals circadian control of splicing events in *Arabidopsis thaliana*. *The* *Plant Journal* **103:** 889-902.

**Rosli HG, Zheng Y, Pombo MA, Zhong S, Bombarely A, Fei Z, Collmer A, Martin GB. 2013.** Transcriptomics-based screen for genes induced by flagellin and repressed by pathogen effectors identifies a cell wall-associated kinase involved in plant immunity. *Genome Biology* **14:** 1-15.

**Parkinson H, Kapushesky M, Shojatalab M, Abeygunawardena N, Coulson R, Farne A, Holloway E, Kolesnykov N, Lilja P, Lukk M, et al. 2007.** ArrayExpress--a public database of microarray experiments and gene expression profiles. *Nucleic Acids Research* **35**(Database issue): D747-750.

**Pfaffl MW. 2001.** A new mathematical model for relative quantification in real-time RT–PCR. *Nucleic Acids Research* **29**(9): e45-e45.

**Ru L, Osorio S, Wang L, Fernie AR, Patrick JW, Ruan Y-L. 2017.** Transcriptomic and metabolomics responses to elevated cell wall invertase activity during tomato fruit set. *Journal of Experimental Botany* **68**(15): 4263-4279.

**Stotz HU, Sawada Y, Shimada Y, Hirai MY, Sasaki E, Krischke M, Brown PD, Saito K, Kamiya Y. 2011.** Role of camalexin, indole glucosinolates, and side chain modification of glucosinolate-derived isothiocyanates in defense of *Arabidopsis* against *Sclerotinia sclerotiorum*. *The* *Plant Journal* **67:** 81-93.

**The Tomato Genome Consortium. 2012.** The tomato genome sequence provides insights into fleshy fruit evolution. *Nature* **485:** 635-641.

**Tanaka S, Brefort T, Neidig N, Djamei A, Kahnt J, Vermerris W, Koenig S, Feussner K, Feussner I, Kahmann R. 2014.** A secreted *Ustilago maydis* effector promotes virulence by targeting anthocyanin biosynthesis in maize. *eLife* **3:** e01355.

**Xu Q, Chen S, Yunjuan R, Chen S, Liesche J. 2018.** Regulation of sucrose transporters and phloem loading in response to environmental cues. *Plant Physiology* **176**(1): 930-945.

**Yang N, Lu Y, Yang X, Huang J, Zhou Y, Ali F, Wen W, Liu J, Li J, Yan J. 2014.** Genome wide association studies using a new nonparametric model reveal the genetic architecture of 17 agronomic traits in an enlarged maize association panel. *PLOS Genetics* **10**(9): e1004573.

**Yang Y-X, Wang M-M, Yin Y-L, Onac E, Zhou G-F, Peng S, Xia X-J, Shi K, Yu J-Q, Zhou Y-H. 2015.** RNA-seq analysis reveals the role of red light in resistance against *Pseudomonas syringae pv*. tomato DC3000 in tomato plants. *BMC Genomics* **16:** 1-16.

**Zhang X, Henriques R, Lin S-S, Niu Q-W, Chua N-H. 2006.** Agrobacterium-mediated transformation of *Arabidopsis thaliana* using the floral dip method. *Nature Protocols* **1**(2): 641-646.
